# Quantifying Dispersal of European *Culicoides* (Diptera: Ceratopogonidae) Vectors between Farms Using a Novel Mark-Release-Recapture Technique

**DOI:** 10.1371/journal.pone.0061269

**Published:** 2013-04-22

**Authors:** Carsten Kirkeby, René Bødker, Anders Stockmarr, Peter Lind, Peter M. H. Heegaard

**Affiliations:** 1 Section of Epidemiology, National Veterinary Institute, Technical University of Denmark, Frederiksberg C, Denmark; 2 Department of Applied Mathematics and Computer Science, DTU Compute, Technical University of Denmark, Lyngby, Denmark; 3 Section for Immunology and Vaccinology, National Veterinary Institute, Technical University of Denmark, Frederiksberg C, Denmark; University of Georgia, United States of America

## Abstract

Studying the dispersal of small flying insects such as *Culicoides* constitutes a great challenge due to huge population sizes and lack of a method to efficiently mark and objectively detect many specimens at a time. We here describe a novel mark-release-recapture method for *Culicoides* in the field using fluorescein isothiocyanate (FITC) as marking agent without anaesthesia. Using a plate scanner, this detection technique can be used to analyse thousands of individual *Culicoides* specimens per day at a reasonable cost. We marked and released an estimated 853 specimens of the Pulicaris group and 607 specimens of the Obsoletus group on a cattle farm in Denmark. An estimated 9,090 (8,918–9,260) Obsoletus group specimens and 14,272 (14,194–14,448) Pulicaris group specimens were captured in the surroundings and subsequently analysed. Two (0.3%) Obsoletus group specimens and 28 (4.6%) Pulicaris group specimens were recaptured. The two recaptured Obsoletus group specimens were caught at the release point on the night following release. Eight (29%) of the recaptured Pulicaris group specimens were caught at a pig farm 1,750 m upwind from the release point. Five of these were recaptured on the night following release and the three other were recaptured on the second night after release. This is the first time that movement of *Culicoides* vectors between farms in Europe has been directly quantified. The findings suggest an extensive and rapid exchange of disease vectors between farms. Rapid movement of vectors between neighboring farms may explain the the high rate of spatial spread of Schmallenberg and bluetongue virus (BTV) in northern Europe.

## Introduction

Vector-borne diseases are of great concern in all parts of the world. In northern Europe, incoming disease agents such as bluetongue virus and Schmallenberg virus have recently appeared where *Culicoides* borne diseases have previously not been a problem (e.g. [1], [2]). Epidemiological models for the spread of vector-borne diseases such as bluetongue virus rely on accurate data describing the underlying mechanisms [3]–[5]. Especially the dispersal distance, speed and direction is of high importance when simulating outbreaks of vector-borne diseases [5]–[7].

Mark-release-recapture (MRR) techniques have been used in many studies to investigate the behavior of different insects, e.g. beetles [8], grasshoppers [9], flies [10], termites [11], mosquitoes [12] and fruit flies [13]. In MRR studies, it is necessary to mark a relatively large proportion of the population because the propability of recapture can be very low as a result of mortality and emigration. The number of *Culicoides* specimens at a location can be enormous in some places, reaching over a thousand specimens caught in a single trap [1]. Thus MRR studies of *Culicoides* requires a high number of marked specimens and high-throughput detection. It also requires a sensitive detection technique because of their small size.

Very few MRR studies have been conducted on *Culicoides* previously:

In 1977, Lillie et al. [14] anaesthetized, marked and released 82,200 specimens of *Culicoides variipennis* with micronized fluorescent dust in Denver, Colorado. 403 marked specimens were recaptured in CO_2_-baited traps. Recaptured specimens were detected by eye inspection under UV-light. They found one female that had dispersed 4 km in 36 hours.

Brenner et al. [15] studied *C. mohave* in the desert of Southern California in 1981. Traps were baited with dry ice. In the marking procedure, specimens were anaesthetized with CO_2_ and shaken in a container with fluorescent powder. Marked specimens were detected by examination under UV-light on a black background. In that study, almost 14% of 20,646 marked specimens were recaptured. They found that most specimens dispersed downwind but also found a female 6 km upwind 30 hours after release. They further speculated that *Culicoides* exhibit omnidirectional flight rather than either upwind or downwind dispersal, although most specimens in this study were caught downwind.

In 1984, Lillie et al. [16] conducted a study where 40,000 specimens of *Culicoides mississippiensis* were marked and released. In this study no anaesthetization was used and *Culicoides* were caught in CDC light traps baited with CO_2_. During two-four day periods following two releases, 567 (1.4%) specimens were recaptured up to 3.2 km away from the release point. At this position a single specimen was caught 24 hours after release. There were no indications of influence of wind direction on the flight direction in this study.

According to Hagler & Jackson [17], an ideal marker for insects is “durable, inexpensive, nontoxic, easily applied, and clearly identifiable”. Until now, MRR studies of *Culicoides* have been based on subjective visual eye inspection to detect marked specimens under UV light. Here we take a new approach and use a novel method for marking *Culicoides* with an objective method of detection of marked specimens.

Most models for the spread of bluetongue virus assume that vectors fly in random directions and can be transported with the wind over long distances. Recently, Sedda et al. [7] developed a model to simulate the 2006 outbreak of BTV in northern Europe including upwind flight of the vectors. They found that downwind flight, as included in previous models, was not sufficient to explain the number of infected farms. Thus they included upwind flight and mixed random flight, and were able to explain 94% of all observed farm infections. They concluded that upwind flight of the vectors was responsible for 38% of the infections. In this study we directly quantify the dispersal of European *Culicoides* vectors between farms for the first time.

## Results

### Method validation results

The fluorescence cutoff value between negative (unmarked) and positive (marked) specimens were defined as the mean of the negative controls, consisting of the mean of two scans, plus five times the standard deviation of those values. The mean value was 45, and the standard deviation 18.5, and thus the cutoff for negative measurements was 138 for the described scanning conditions. We used the mean value of two scans as a measure of fluorescence, which resulted in 30 specimens with a mean value higher than the cutoff. The correlation between the first and the second scan for the negative specimens was 0.65, and for the positive specimens 0.996.

The mean of the measured fluorescence emission of the laboratory marked specimens in the carryover study were approximately ten fold higher (minimum: 9,323) than the marked and recaptured specimens in the field (maximum: 1,701). The ranges of the scanned value of negative wells and the wells that were neighbours to a well with marked specimen overlapped and thus we did not test this further. No cross-staining between specimens or contamination from tweezers was detected (data not shown).

### Field study results

**Figure 1:**
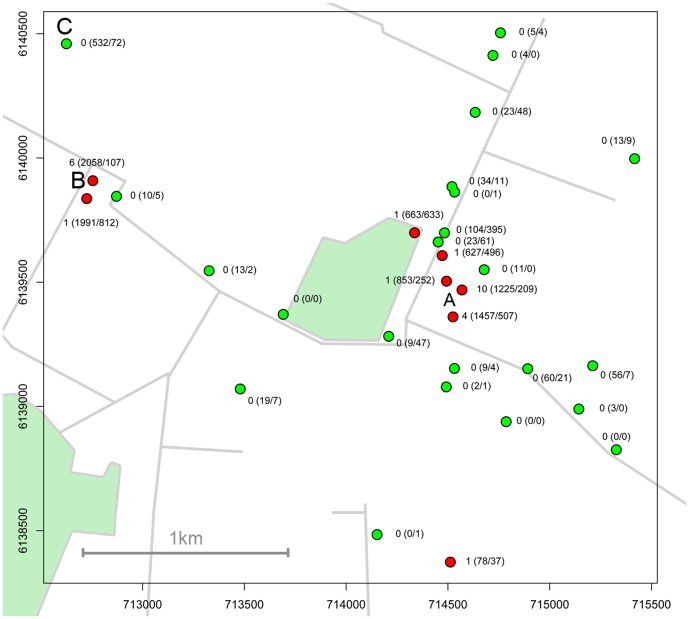
The spatial distribution of the trap catches in the first period in the study (July 22*^nd^*–July 27*^th^*). Axes represent the UTM coordinates. The dots represent the trap locations and red dots are locations where Pulicaris specimens were recaptured. The numbers at each location represent for this period: Pulicaris group specimens recaptured (Pulicaris group specimens caught/Obsoletus group specimens caught). The letters show locations of the release point of marked *Culicoides* where 700 cattle were stabled (A), the 1,700 pigs (B) and the 20 angus cattle (C).

**Figure 2:**
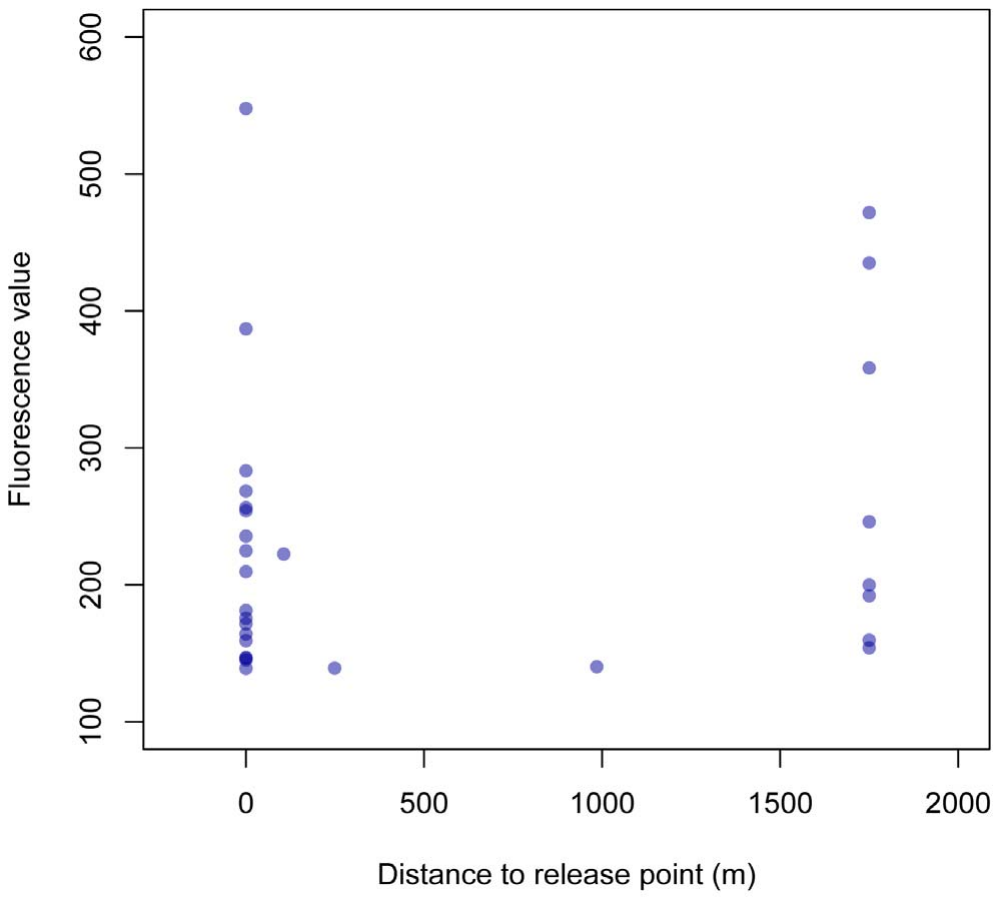
The mean fluorescence value for each recaptured specimen plotted against the dispersal distance. One specimen with fluorescence value = 1,701 recaptured in the release point is not shown. The values of the specimens recaptured at 1,750 m from the release point are similar to those recaptured in the release point. Increasing the cutoff value did not affect the ratio between recaptured specimens at zero and 1,750 m distance to the release point.

An estimated 607 Obsoletus group and 853 Pulicaris group specimens were marked and released at the study site (Fig. 1), and an estimated 9,090 female Obsoletus group and 14,272 female Pulicaris group specimens were caught during the study period (Table 1). Of these, two females (0.3%) of the marked Obsoletus group specimens and 28 females (3.3%) of the marked Pulicaris group specimens were recaptured. This yields a total recapture percentage of 2.1% (30/1460). The mean of fluorescence values of the marked specimens was 264, ranging from 142 to 1,701. The fluorescence values and recapture distance from the release point is shown in Fig. 2. The two recaptured Obsoletus group specimens were both caught in the first marking period where it was estimated that only 96 Obsoletus group specimens were marked (Table 1).

**Table 1:** Results For each marking period in the study: The estimated number of marked specimens (recaptured specimens in parentheses); the number of captured specimens (95% C.I.); the number of trap catches; the mean number of specimens per trap catch; the mean (minimum and maximum) wind speed; and the mean temperature (minimum and maximum) measured during the four study periods.

	Marked (recaptured)		Captured (95% C.I.)		
Period	Obsoletus	Pulicaris	Obso.	Puli.	Trap catches
P1	96 (2)	274 (25)	3749 (3645–3851)	9882 (9768–9996)	189
P2	234 (0)	150 (1)	2931 (2884–2978)	2931 (2986–2976)	391
P3	222 (0)	378 (2)	1829 (1818–1840)	1110 (1100–1118)	236
P4	21 (0)	15 (0)	581 (571–591)	349 (340–358)	284
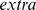	34	136			
**Total**	607 (2)	853 (28)	9,090	14,272	1110

	Mean catch per trap			
Period	Obsoletus	Pulicaris	Wind speed (mean m/s)	Temperature (mean °C)
P1	20	52	1.4 (0–4.5)	15.2 (10.4–20.1)
P2	7	7	1.2 (0–3.1)	16.0 (11.7–20.3)
P3	5	5	0.7 (0–3.6)	15.6 (8.1–19.6)
P4	2	1	0.8 (0–2.7)	15.2 (8.7–20.9)

Weather variables are measured during the *Culicoides* active periods.

The two recaptured Obsoletus group specimens were caught in a trap at the release point for marked specimens. They were caught on the first night in the first marking period, meaning that they had been marked for maximum 24 h before recapture.

An overview of the results of the first release period is shown in Fig. 1. In the first marking period, 25 specimens of the Pulicaris group were recaptured out of an estimated 274 marked specimens. In the second release period only one Pulicaris group specimen was recaptured at the pig farm on the second night after release. In the third release period two Pulicaris group specimens were recaptured in the release point. In the fourth release period no marked specimens were recaptured.

In total, 18 of the Pulicaris specimens were recaptured on the first night after release; nine specimens were recaptured on the second night after release; and one specimen was recaptured four nights after release. Eight (29%) of the recaptured Pulicaris group specimens were caught on the pig farm at 1,750 m distance from the release points of marked specimens. Of these eight specimens, five (63%) were recaptured on the neighboring pig farm one day after release, having dispersed 1,750 m in less than 24 hours. The last three (38%) of the eight specimens were caught at the pig farm on the second night after release. From the Pulicaris group, 17 (61%) of the recaptured specimens were caught in the traps at release points of marked specimens. A single Pulicaris group specimen was recaptured after one night in a trap 250 m north-west of the release point; and two Pulicaris group specimens were caught on the second night after release, one in a trap 100 m north-west of the release point and the other one in a trap 1 km south of the release point (Fig. 1). During the whole study period the mean number of specimens caught per trap declined for both species groups, indicating that the abundance was declining (Table 1).

Because there exists no gold standard test that can be used to evaluate the cutoff, we also removed half of the specimens with the lowest half of the mean fluorescence values from the data. This was to test if the specimens caught on the pig farm had low fluorescence values. Using this high cut off, again 29% (4 out of 14) Pulicaris group specimens were recaptured on the pig farm. The fluorescence values are shown in Fig. 2.

Weather variables were measured during the whole study. All values presented are measured during the *Culicoides* active periods, which we defined to be one hour before to three hours after sunset and two hours before to one hour after sunrise. The wind direction was predominantly from west during all four study periods. In the first period the wind blew mostly from west and north-west; in the second period it blew from south-west; in the third period it blew from north-west; and in the fourth period it blew from south-west and north-west. The mean wind speed was declining during the four periods, going from 1.4 to 0.8 m/s (Table 1). Also the maximum wind speeds measured declined during the study period, going from 5.4 to 2.7 m/s. The mean temperature did not change much during the study period, but the minimum temperature in the *Culicoides* active periods went from 10.4 to 8.7°C (Table 1).

## Discussion

We have here presented and tested a novel technique to mark and recapture *Culicoides* in the field and subsequently scan them individually. We have only used the technique for quantifying the proportion of marked specimens moving from one location to another. If the technique should be used for e.g. survival rate studies, more tests are needed, for instance how fast the light-sensitive FITC fades in nature. We have also not tested the impact of the marking method on the survival rate of marked specimens.

Most models for the spread of bluetongue assumes random local flight of the vectors [5], [6], [18], [19].

In this study we found that 29% (8/28) of the recaptured Pulicaris specimens were recaptured at the pig farm, indicating that vectors actively disperse upwind to seek hosts like e.g. female host-seeking mosquitoes [20]. This is in contrast to the findings of Brenner et al. [15] who found that marked specimens of *C. mohave* dispersed omnidirectionally but mostly downwind. However, in that study a single female was recaptured 6 km upwind after 30 hours. Bhasin et al. [21] found that females of *C. impunctatus* showed upwind flight towards plumes of CO_2_. Our findings supports the intense upwind dispersal, which Sedda et al. [7] found responsible for 54% of the infected farms in 2006. In that study, it was assumed that vectors could detect the odor of neighboring farms at a maximum distance of 300 m. Our results indicate that this distance is at least 1,750 m for the Pulicaris group. This is, to our knowledge, the first time that dispersal of European *Culicoides* vectors have been quantified between farms. The described measures of speed, distance and direction related to wind is useful when modeling the spread of e.g. bluetongue and Schmallenberg virus. However, we were not able to recapture more than two Obsoletus group specimens, the supposed main vector for BTV in northern Europe [2], and thus further studies are needed to investigate the dispersal pattern for this species group. In future studies it will also be relevant to address if FITC has an impact on the mortality of the marked specimens. Perhaps the low number of recaptured Obsoletus group specimens is caused by increased mortality for this species group when marked with FITC.

In 2008, when BTV was present in Denmark and other countries in northern Europe, 97.5% of the Danish cattle farms were placed within 1600 m distance of the nearest cattle farm (Kaare Græ sbøllpers. comm.). Thus the results of this study suggest that vectors are capable of transmitting disease between almost all Danish farms very efficiently.

The sensitivity of the present technique is potentially higher than in previous studies [15], [16] because the scanning procedure used in this study can detect very small amounts of FITC. An advantage of the present technique is also that the insects can be marked without anaesthetisation, unlike some previous studies [15], [16]. By marking live specimens, mortality and morbidity of the insects due to anaesthesia is avoided and their behavior is likely less interrupted. Furthermore, the detection of marked specimens in this study does not rely on subjective judgement of whether a specimen is marked or not.

When setting up field experiments for small flying insects such as *Culicoides*, weather conditions will influence the catch numbers greatly [22], [23]. The more specimens that are marked, the greater the possibility of recapture. Thus it can be necessary to boost the number of marked specimens caught at other locations, as we did in the last period of this study. However, we marked relatively few individuals during this study, compared to the total number of specimens caught, and this would be an obvious place to improve a future setup, e.g. by baiting traps with CO_2_ when catching specimens for marking.

In the present study we recaptured 2.1% (30/1460) of the marked specimens. This number is higher than found in Lillie et al. [14] where 0.49% (403/82,200) were recovered, and in Lillie et al. [16] where 1.5% (498/25,000) were recovered, but lower than the study of Brenner et al. [15] where almost 14% (2794/20,646) of marked specimens were recaptured. As speculated in Lillie et al. [16], the higher recapture percentage of *C. mohave* [15] could be caused by the desert environment lacking obstacles to obstruct the attraction of the traps. We further speculate that the hostile desert environment where *C. mohave* lives can cause specimens to actively search more for breeding sites or host animals and thus make traps more efficient.

In this study we recaptured 29% of the Pulicaris group specimens on the pig farm 1,750 m away from the release point (Fig. 1). We tested if the recaptured specimens here had lower fluorescence values than those recaptured in a release point. Removing the lower half of the fluorescence values from the data had no effect on the estimated relative dispersal, indicating that the selected cutoff was robust. Thus the specimens recaptured on the pig farm are regarded as true positives.

The two Obsoletus group specimens recaptured in this study were caught in the same location as they were released. Although more recaptures are needed to investigate their dispersal behavior thoroughly, it may reflect a general pattern: As stated in Marquardt et al. [24], species of Ceratopogonidae that breed in temporary habitats tend to disperse more broadly than species that breed in more permanent habitats. As showed by Zimmer et al. [25] and Ninio et al. [26], species of the Obsoletus group breed in dung and manure inside stables. These breeding sites are more permanent and location-specific than temporary water bodies where the Pulicaris group breed [27]–[29]. Thus there may be different dispersal patterns for the two species groups.

A concern in this study was that the specimens would die or no specimens would be recaptured during the study, which is why we chose to mark four times instead of one. The drawback of this approach is that we cannot determine if recaptured specimens in the second, third and fourth periods were marked in the same period they were caught. In this study we assumed that recaptured specimens were released on the nearest release date before recapture. However, it would be more optimal to mark and release only one time during a study period.

An unknown factor in this study is that the *Culicoides* can get in contact with everything in the study area before recapture. If e.g. some types of pollen exhibit autofluorescence, this can cause noise in the data. This is a potential source of bias. In the present study we used unmarked specimens from the study site to establish a cutoff between marked and unmarked specimens. If a source of pollution introduce fluorescence, this will be adjusted for in the cutoff. However, it will also cause weakly marked specimens to be unregistered because their fluorescence will be less than the cutoff.

From the present field experiment it is evident that the vector abundance is higher near host animals (Fig. 1). Traps that are placed far from hosts on agricultural land caught less *Culicoides* than traps near hosts. This conforms with the findings of Rigot et al. [30] who found decreasing numbers of *Culicoides* associated with farms when distance to farms increased.

The present technique is a novel tool for the investigation of the dispersal of small flying insects such as *Culicoides*. It has great potential for estimating important parameters for epidemiological models for vector-borne diseases, such as migration between farms as described in the model of Hanski et al. [31], population size as in Trpis et al. [32] and survival rate like Rosewell et al. [33].

## Materials and Methods

### Ethics statement

The trap locations in the field experiment were placed on private property. All land owners were contacted before the field experiment, and all traps were set up according to permission from the land owners. The field work did not involve any endangered or protected species.

### Marking method

Fluorescein is an orange staining dye commonly used in microscopy. If excited with fluorescent light at approx. 494 nm, it emits light at approx. 521 nm and is therefore a useful tool in ELISA plate scanning. Fluorescein isothiocyanate (FITC) is fluorescein with a reactive SCN group (thiocyanate), used previously to label chitinase [34]. FITC in powder form must be kept in a dark container in order not to fade, but is otherwise stable.

We used FITC powder in this study to mark the specimens. The amount of powder that can adhere to small specimens of *Culicoides* is of course small, making detection with the naked eye difficult. Therefore we used a Tecan SpectraFluor Plus plate scanner and the Xfluor software (www.tecan.com) for detection of FITC on specimens. To each well in ELISA plates with flat bottom were added 100
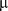
L 70% ethanol to extract the FITC and preserve the *Culicoides*. It also removed most of the static electricity which could make it difficult to place the dry specimens in the wells. All plates, with one specimen of *Culicoides* in each well, were gently shaken on a shaking table for five minutes prior to scanning. The plates were then scanned in the Tecan scanner with excitation wavelength set to 485 nm and emission wavelength set to 530 nm. Gain was set to 55 in all trials and measurements were carried out with three flashes, 0 s lag time, 40
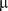
s integration time and an initial 10 s shake to distribute dissolved FITC in the ethanol. All plates were scanned twice, to increase the precision of detection. About 25 plates could be scanned in one hour. After scanning, the resulting data files were run through an automated procedure in R 2.14.2 (R Development Core Team, 2011), screening for measured values higher than a defined cutoff level.

To identify a cutoff level for unmarked specimens, 192 *Culicoides* from the field experiment (see below), caught on the day before marking experiments started, were scanned twice using the scanning procedure. In order to exclude false positive specimens from the data, the cutoff was set to mean 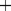
5*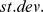 Assuming a normal distribution and using this level, only one in 1.7 million specimens will be false positive. At this cutoff level some marked specimens are likely to be undetected and wrongly classified as negative, but the priority in this study was to avoid any false positives because false negatives do not affect the proportional estimates of dispersal.

To validate the method we tested for cross-staining, laboratory contamination and carryover of emitted light between wells. We marked dead specimens by shaking them in a beaker with FITC powder. They were then transferred to a clean beaker with unmarked dead specimens and shaken for one minute. To test for contamination from using the same tweezers to handle marked and unmarked insects, we placed ten lab marked specimens in a plate and subsequently used the same tweezers to place six unmarked specimens.

There was a potential risk of a carryover effect of fluorescent light from a marked specimen in a well, to neighboring wells in the same plate with unmarked specimens, because regular transparent ELISA plates were used. To test this, dead specimens of *Culicoides* were marked by shaking them in a beaker with FITC. Then five of these marked specimens were put into the wells on a plate with unmarked neighbors using the procedure as described above. The plate was then scanned as in the procedure described above.

### Field experiment

The field experiment was conducted between July 21 and August 14, 2010, on a study farm in Denmark (geographical coordinates: N55.35477, E12.381). This farm was chosen because the nearest farm was 1,750 m away which is a large distance in Denmark. The entire stable walls and the sliding doors in the ends were open, allowing *Culicoides* to freely enter and leave. The nearest farms were a small outdoor angus cattle holding with 20 outdoor animals at a distance of 2.0 km (West-North-West of the study farm) and a pig farm with about 1,700 animals indoors at a distance of 1.75 km (West of the study farm, see Fig. 1). The odor of pigs was emitted from the pig farm through a ventilation system. We also checked that no host animals that might attract *Culicoides* were present in other locations in the study area. During the study period a weather station (Davis Vantage Pro 2) measured the wind direction and temperature in 10 min intervals. The weather station was set up in the study area more than 100 m from any trees that could obstruct the wind. Supplementary data on the wind direction from an official weather station 10 km from the release point (Danish Meteorological Institute) was used in periods when the local weather station was not working.

Breeding sites for *Culicoides* were distributed throughout the study area. For the Obsoletus group, potential breeding sites were in leaf litter and decaying wood in forest areas primarily 400 m east of the farm, dung in the stables and a big dunghill next to the stables. Potential breeding sites for the Pulicaris group were present on surrounding fields around small ponds and marl pits [27]–[29].

Around the study farm, 45 traps were set up in locations approximating four transects out from from the farm (see Fig. 1). On the pig farm 1,750 m west of the study farm (and release point), two groups of three traps each were hung up side by side near the stable, assuming that the abundance of *Culicoides* would be high here, and that *Culicoides* from the release points might disperse towards the pig farm. The trap type used was the CDC New Standard Miniature 4 W Blacklight Trap (Model 1212, www.johnwhock.com) using a 6 V battery and equipped with a photoswitch that automatically turned the trap on at dusk and off at dawn. Traps were hung up in a height of approximately 180 cm, on the stable wall, in branches on windbreaks where available and otherwise in heavy metal gallows constructed for the purpose. In each of three locations on the study farm, four traps were hung up side by side on the stable walls. At each of these three locations, trap catches were marked and released. The *Culicoides* were not anaesthetised upon marking, hence the number of marked specimens could not be counted directly. Therefore, to estimate the number of individuals marked and released, the specimens caught in the fourth trap was killed and preserved in 70% ethanol. We assumed that this trap caught 1/4 of the total catch in each location, which was the general pattern observed on the catch nights where all four traps were killed and analysed. On the 07. August, extra *Culicoides*, caught at a farm 3 km away (geographical coordinates: N55.3619, E12.3234), were released together with the other released specimens on the same day, in order to increase the number of marked specimens. The number of released specimens from this location was estimated by another trap catching *Culicoides* side by side with the marked trap (Table 1).

Before the study, a schedule was set up for marking specimens on the study farm once a week to allow marked specimens to disperse between markings. However, if low numbers of *Culicoides* were caught on the night planned for marking, it was postponed to the next night with catches high enough for feasible marking. We succeeded to mark *Culicoides* on four different dates during the study period with minimum five days between markings. We marked specimens in the morning of the July 22*^nd^*, July 27*^th^*, August 1*^st^* and August 7*^th^*. The periods between markings and until August 14*^th^* after the last marking date are referred to as the marking periods (P1–P4 in Table 1).

The marking was carried out in the morning at the locations where the specimens were caught, using the following procedure: A flow of air was created with a dust blower commonly used to clean camera lenses (InnoDesk, Inc., Cleveland, Ohio, USA). The dust blower runs on batteries so it can be used in the field, and creates a moderate consistent stream of air just enough to make a cloud of powder particles but not enough to kill the *Culicoides*. The air was led through a 50 cm long and 0.6 mm wide plastic tube into a small (9 cm, 38 mm diameter) closed beaker containing approx. 5 ml FITC. In this beaker, the FITC powder was mixed with air into a dust cloud. From the beaker, the dust cloud was lead further through another 50 cm long and 0.6 mm wide plastic tube into a 500 ml beaker with the caught insects. The plastic tube entered the beaker through a hole in the lid, and the air stream escaped through another hole covered with a fine mesh. The insects were gently swirled around in the flow of air for approx. 5 seconds, ensuring that all specimens had been in contact with the orange marking powder. After marking, insects were released onto the ground at the catch site. Plastic gloves were worn at all times when marking, and all marking equipment was carefully packed separately from other equipment to avoid contamination.

All caught *Culicoides* that were not marked and released were killed quickly with a small piece of paper stained with ethyl acetate. They were then stored at −20°C. Only a subsample of each trap catch was morphologically identified, following Campbell & Pelham-Clinton [35]. If containing more than 20 specimens, catches were subsampled according to the Raosoft sample size calculator (www.raosoft.com/samplesize.html) using 5% error margin and a confidence level of 95%. Females were then transferred to an ELISA plate with one specimen per well. This was the most time-consuming step in the procedure, and allowed all specimens to be scanned individually. Each plate was scanned twice in the Tecan scanner, and the mean value of the two scans was used as the measure of fluorescence. All positive specimens were identified to species group level.
